# Hijacking of the jasmonate pathway by the mycotoxin fumonisin B1 (FB1) to initiate programmed cell death in *Arabidopsis* is modulated by RGLG3 and RGLG4

**DOI:** 10.1093/jxb/erv068

**Published:** 2015-03-18

**Authors:** Xu Zhang, Qian Wu, Shao Cui, Jiao Ren, Wanqiang Qian, Yang Yang, Shanping He, Jinfang Chu, Xiaohong Sun, Cunyu Yan, Xiangchun Yu, Chengcai An

**Affiliations:** ^1^The State Key Laboratory of Protein and Plant Gene Research, College of Life Sciences, Peking University, Beijing 100871, China; ^2^National Center for Plant Gene Research (Beijing), Institute of Genetics and Developmental Biology, Chinese Academy of Sciences, Beijing 100101, China; ^3^ Present address: Basic Research Service, Ministry of Science and Technology of the People’s Republic of China, 15B, Fuxing Road, Beijing 100862, China; ^4^ Present address: Great Lakes Bioenergy Research Center, Michigan State University, East Lansing, MI 48824, USA; ^5^ Present address: Department of Molecular Microbiology and Immunology, University of Southern California, Los Angeles, CA 90089, USA

**Keywords:** *Arabidopsis*, fumonisin B1, jasmonate, programmed cell death, RGLG3, RGLG4, salicylic acid.

## Abstract

The mycotoxin fumonisin B1 (FB1) is a strong inducer of programmed cell death (PCD) in plants, but its underlying mechanism remains unclear. Here, we describe two ubiquitin ligases, RING DOMAIN LIGASE3 (RGLG3) and RGLG4, which control FB1-triggered PCD by modulating the jasmonate (JA) signalling pathway in *Arabidopsis thaliana*. *RGLG3* and *RGLG4* transcription was sensitive to FB1. *Arabidopsis* FB1 sensitivity was suppressed by loss of function of RGLG3 and RGLG4 and was increased by their overexpression. Thus RGLG3 and RGLG4 have coordinated and positive roles in FB1-elicited PCD. Mutated JA perception by *coi1* disrupted the *RGLG3*- and *RGLG4*-related response to FB1 and interfered with their roles in cell death. Although FB1 induced JA-responsive defence genes, it repressed growth-related, as well as JA biosynthesis-related, genes. Consistently, FB1 application reduced JA content in wild-type plants. Furthermore, exogenously applied salicylic acid additively suppressed JA signalling with FB1 treatment, suggesting that FB1-induced salicylic acid inhibits the JA pathway during this process. All of these effects were attenuated in *rglg3 rglg4* plants. Altogether, these data suggest that the JA pathway is hijacked by the toxin FB1 to elicit PCD, which is coordinated by *Arabidopsis* RGLG3 and RGLG4.

## Highlight

Two ubiquitin ligases control fumonisin B1-elicited programmed cell death by modulating jasmonate signalling transduction in *Arabidopsis*.

## Introduction

Programmed cell death (PCD) is an essential biological process invoked during normal growth and development or under conditions of stress (Lam, 2004; Dickman and Fluhr, 2013). Several types of PCD exist in plants, and many PCD-related morphologies have been observed, such as cell volume reduction, chromatin condensation and nuclear segmentation (Reape and McCabe, 2008; van Doorn *et al.*, 2011). A widely studied form of plant PCD is the hypersensitive response (HR), which takes place during incompatible pathogen-plant interactions and during which plant cells around the invasion site(s) actively and rapidly die to stop the supply of nutrients and limit pathogen growth, thereby preventing the disease from spreading throughout the whole plant (Coll *et al.*, 2011).

To evade the host plant immune system, many pathogens, most of which are biotrophic, have evolved mechanisms to suppress the host plant HR by delivering specific effectors into the infected cell (Jamir *et al.*, 2004; Bos *et al.*, 2006; Kelley *et al.*, 2010). However, a number of bacterial and fungal pathogens, most of which are necrotrophic, promote cell death as a source of nutrients from dead or dying cells by secreting toxins in the host plant (Howlett, 2006). Fumonisin B1 (FB1) is a potent sphingolipid-like PCD elicitor produced by the compatible fungal pathogen *Fusarium moniliforme* that causes serious disease symptoms in maize and other grains (Gilchrist, 1997, 1998). FB1 inhibits sphingolipid biosynthesis by competitive inhibition of ceramide synthase, which can lead to cell death (Stone *et al.*, 2000; Desai *et al.*, 2002), and, in *Arabidopsis*, depletes extracellular ATP (Chivasa *et al.*, 2005). In addition to cell death, FB1 can elicit other HR-like responses including reactive oxygen species (ROS) generation, phenolic compound and callose deposition, phytoalexin accumulation and pathogenesis-related (PR) protein expression (Wolpert *et al.*, 2002). FB1-induced cell death thus provides a simple pathogen-free system for elucidating the molecular basis of HR (Stone *et al.*, 2000). However, the mechanism underlying FB1-triggered PCD is not well understood.

Jasmonates (JAs) are essential hormones that mainly mediate plant defence responses to necrotrophic pathogens (Glazebrook, 2005). In the inactivated state, jasmonate zim-domain (JAZ) proteins suppress the JA pathway by binding to various transcription factors (Boter *et al.*, 2004; Lorenzo *et al.*, 2004; Dombrecht *et al.*, 2007; Fernandez-Calvo *et al.*, 2011; Qi *et al.*, 2011; Song *et al.*, 2011; Kazan and Manners, 2012). Pathological stress promotes rapid JA production through the consecutive actions of diverse synthesis enzymes such as allene oxide synthase (AOS), OPDA reductase 3 (OPR3) and OPC-8:0 CoA ligase 1 (OPCL1) (Browse, 2009). JA can be further modified into numerous conjugates including the highly bioactive (+)-7-iso-jasmonoyl-l-isoleucine (JA-Ile) (Staswick and Tiryaki, 2004; Fonseca *et al.*, 2009; Thines *et al.*, 2007). JA-Ile is recognized by a receptor complex consisting of coronatine insensitive 1 (COI1), JAZs, and inositol pentakisphosphate (InsP5) (Sheard *et al.*, 2010), which promotes COI1 to form a large SKP/CUL/F-box complex (SCF^COI1^) with other partners (Devoto *et al.*, 2002; Xu *et al.*, 2002) and then targets JAZs for degradation by the 26S proteasome, thus activating downstream signalling (Chini *et al.*, 2007; Thines *et al.*, 2007).

The contribution of the JA pathway to PCD remains inconclusive, although several studies have provided conflicting results. For example, methyl jasmonate (MeJA) pretreatment of *Arabidopsis* ecotype Col-0 abolishes O^3^-induced cell death, and the JA-insensitive mutants *jar1* and *fad3/7/8* become more sensitive to O^3^ than wild type, suggesting that the JA pathway has a negative role in O^3^-induced PCD (Overmyer *et al.*, 2000; Rao *et al.*, 2000). In contrast, FB1-treated *Arabidopsis* protoplasts prepared from *jar1* plants display enhanced viability (Asai *et al.*, 2000), and, in *Nicotiana benthamiana*, interrupting the JA pathway can affect the positive role of NbHB1 in pathogen-induced cell death (Yoon *et al.*, 2009), indicating a positive role for the JA pathway in PCD. Thus the molecular mechanism of JA in PCD awaits further studies.

Despite previous studies in *Arabidopsis* protoplasts indicating that FB1-induced PCD requires salicylic acid (SA), JA and ethylene (ET) signalling pathways (Asai *et al.*, 2000), the actual roles of these hormone pathways have not been determined in plants. We have studied the functions of two recently identified JA pathway regulators, RGLG3 and RGLG4 (Zhang *et al.*, 2012), in FB1-elicited PCD. Our in-plant analysis showed that a functional JA pathway was required for FB1 to trigger PCD. Despite that, FB1 suppressed JA production and part of the JA signalling pathway. Exogenous SA application promoted FB1 suppression of JA signalling, suggesting that FB1-induced SA could further inhibit the JA pathway. Significantly, these effects required the involvement of both RGLG3 and RGLG4. We propose that the JA pathway is hijacked by FB1 to initiate PCD, and RGLG3 and RGLG4 act as essential coordinators of this process.

## Materials and methods

### Plant materials and growth conditions

*Arabidopsis* ecotypes of Columbia-0 (Col-0) and Columbia glabrous (Col-gl) were used as wild type. Mutants of *rglg3*, *rglg4, rglg3 rglg4, npr1-3*, *ein2-5*, *coi1-1*, *coi1-2*, *myc2-2*, *ein3-1 eil1-1*, the overexpression plants and transgenic materials expressing *promoter::GUS* constructs of RGLG3 and RGLG4 were obtained as previously described (Zhang *et al.*, 2012); *eds5-1* (CS3735) was requested from the Arabidopsis Biological Resource Center. Seeds of Col-gl, *NahG*, *NahG coi1-1, pad4*, *pad4 coi1-1* and *sid2* were requested from Christiane Gatz, *myc2 myc3 myc4* from Roberto Solano and *npr1-1 ein2-1 jar1-1* from Xinnian Dong. All plants were grown under similar conditions, as described (Zhang *et al.*, 2012).

### Protein subcellular localization

Full-length RGLG3 and RGLG4 were amplified by PCR, and each was cloned into pRTL-GFP, generating a construct expressing an N-terminal GFP fusion protein. *Arabidopsis* mesophyll protoplast preparation, transfection and 4,6-diamidino-2-phenylindole (DAPI, 1mg/ml; Sigma, USA) staining were performed as described (He *et al.*, 2011b). Confocal imaging was performed with a Zeiss LSM 710 NLO laser scanning confocal microscope (excitation 488nm; emission 505–550nm). Expression of GFP alone (from the control pRTL-GFP), GFP-RGLG3 and GFP-RGLG4 was confirmed by western blotting using anti-GFP (Abcam, Germany) as the primary antibody and horseradish peroxidase (HRP)-conjugated anti-mouse IgG (Promega, USA) as the secondary antibody.

### Fractionation of cytosolic and nuclear proteins

Cytosolic and nuclear protein extraction was carried out with CELLYTPN1 CelLytic PN isolation/Extraction Kit (Sigma, USA). Briefly, plant tissues were ground in liquid nitrogen and then gently resuspended with 1× nuclei isolation buffer supplemented with 1mM dithiothreitol (DTT), 2mM Na_3_VO_4_, 2mM NaF and 25mM phenylmethylsulfonyl fluoride (PMSF). The resulting suspension was filtered through five layers of Miracloth (Calbiochem, Germany) by centrifugation at 4°C. The pellet was resuspended with 1× nuclei isolation buffer supplemented with 0.3% (v/v) Triton X-100, 1mM DTT, 2mM Na_3_VO_4_, 2mM NaF, 25mM PMSF, 2.5mg/ml antipain, 2.5mg/ml chymostatin, 1mg/ml pepstatin, 5mg/ml leupeptin, 5mg/ml aprotinin and 100mM MG132 (Sigma, USA). After incubation on ice for 15min, extracts were centrifuged at 6800rpm for 5min. The resulting supernatant was transferred to a new tube containing 1× SDS buffer and designated as the cytosolic fraction. The pellet was resuspended in the above buffer and centrifuged and resuspended repeatedly until the pellet appeared slightly grey. The pellet was washed with 1× nuclei isolation buffer supplemented with 1mM DTT, 2mM Na_3_VO_4_, 2mM NaF and 25mM PMSF, dissolved in 1× SDS buffer and designated as the nuclear fraction. The cytosolic and nuclear proteins were loaded onto SDS-PAGE gels in proportion to the volume used in the initial extractions and the resuspension volume of each fraction. Western blotting was performed using anti-FLAG (Sigma, USA), anti-GDPase (Agrisera, Sweden) and anti-histone H3 (Abcam, USA) as the primary antibodies and HRP-conjugated anti-mouse/anti-rabbit IgG (Sigma, USA) as the secondary antibody.

### Chemical treatment

For FB1 treatment, well-expanded leaves (~four weeks old) were infiltrated with 10 µM FB1 (Sigma, USA) in 10mM MgCl_2_ by a needleless syringe; mock-treated leaves were injected with 10mM MgCl_2_. Leaf samples were then collected at the indicated time points. To treat *Arabidopsis* seedlings, five-day-old plants were transferred to new MS plates containing 2 µM FB1 for another six days of growth, with MS plates as the control. FB1-induced phenotypes were classified into three categories (hypersensitive, sensitive and insensitive) and calculated as a percentage of the total plant population as reported (Lin *et al.*, 2008). SA treatment was done by directly spraying 2mM SA (Sigma, USA) diluted in water onto four-week-old plants.

### β-Glucuronidase (GUS) assay

GUS staining was performed as described (Zhang *et al.*, 2012), and GUS activity was determined as reported (Jefferson *et al.*, 1987). Briefly, total protein was extracted with GUS extraction buffer (50mM sodium phosphate buffer, pH 7.5; 10mM β-mercaptoethanol; 0.1% Triton X-100 and 10mM EDTA) and quantified using a protein assay kit (Bio-Rad, USA). Then 50mg total protein was incubated at 37°C for 5min before 1mM 4-methylumbelliferyl-β-d-glucuronide (4-MUG) (Sigma, USA) was added. After 60min, a 100-µl sample was taken and 2.4ml of 0.2M Na_2_CO_3_ was added to terminate the reaction. Each sample was then quantified for absorbance at excitation 365/emission 455 with a fluorospectrophotometer (Hitachi, Japan) to calculate 4-methylumbelliferone (4-MU) production. The final GUS activity was expressed as pmol (4-MU) min^–1^ µg^–1^ (fresh weight).

### Ion conductivity measurements

Ion conductivity was measured as described (He *et al.*, 2011a). Briefly, four-week-old *Arabidopsis* leaves grown under short-day conditions in soil were infiltrated with 10 μM FB1 (Sigma, USA) in 10mM MgCl_2_ or with 10mM MgCl_2_ as the mock treatment. Four leaf disks (6mm diameter) from four plants of each genotype were collected with a cork borer 72h after infiltration, immersed in 25ml deionized water for 45min and subsequently moved into a tube containing 6ml water for measuring ion conductivity using a conductivity meter (Hanna, Italy) at the indicated time points.

### Plant hormone quantification

For JA and SA content measurement, four-week-old plant leaves were injected with 10 μM FB1 in l0mM MgCl_2_, and samples were collected at the indicated time points. Then, 200mg of plant tissues was used for JA and SA quantification as described (Fu *et al.*, 2012).

### Quantitative real-time PCR

Total RNA extraction, cDNA synthesis and real-time PCR were carried out as described (Zhang *et al.*, 2012). Primers used for gene expression analysis are listed in Supplementary Table S1.

## Results

### RGLG3 and RGLG4 are localized in both the cytoplasm and nucleus

**Fig. 1.**
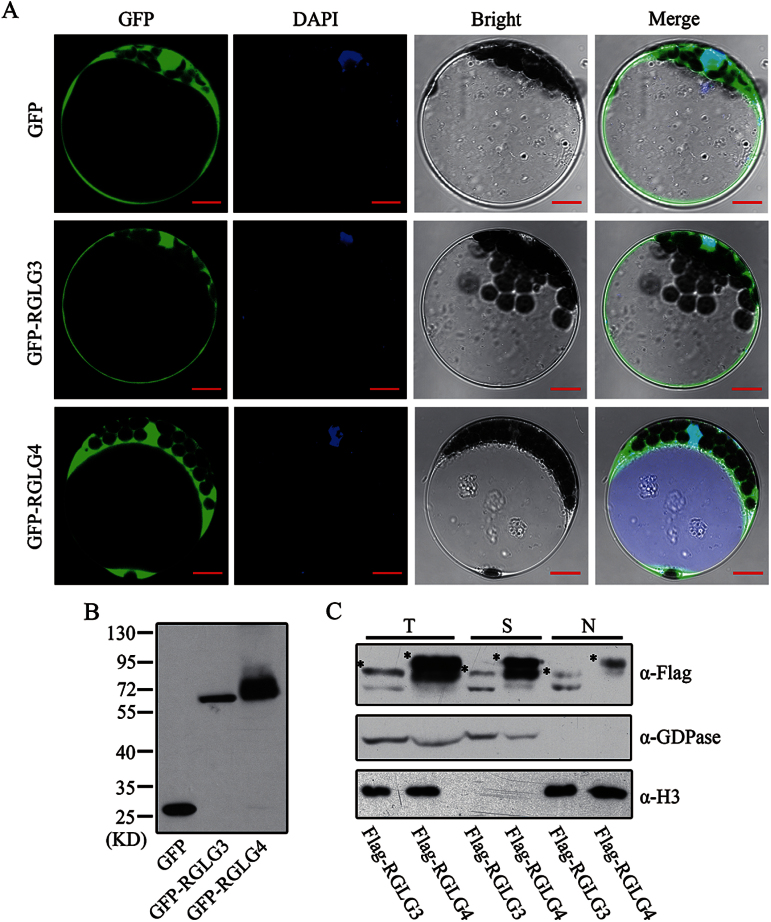
Subcellular localization of RGLG3 and RGLG4. (A) Confocal microscopy images of GFP-tagged RGLG3 and RGLG4 compared with GFP alone expressed in *Arabidopsis* protoplasts. GFP, DAPI, bright field and the merged images of these three signals are shown. Bars, 10 μm. (B) Confirmed expression of GFP, GFP-RGLG3 and GFP-RGLG4 in protoplasts used in (A) by western blotting using GFP antibody. (C) Detection of Flag-tagged RGLG3 and RGLG4 in different subcellular fractions. T, total protein; S, soluble fraction; N, nuclear fraction. H3 antibody was used as a nuclear protein marker and GDPase as a cytoplasmic protein marker. Asterisks indicate positions of FLAG-tagged proteins.

Our recent work has identified two ubiquitin ligases, RGLG3 and RGLG4, as important JA pathway regulators (Zhang *et al.*, 2012). Despite this knowledge, additional properties and possible functions of these two proteins in *Arabidopsis* have awaited further analysis. First, their subcellular distributions remain unclear. We therefore expressed GFP-RGLG3 and GFP-RGLG4 in *Arabidopsis* protoplasts to observe their localization. Confocal images showed that GFP-RGLG3 and GFP-RGLG4 were present in both the cytoplasm and nucleus (indicated by DAPI staining), closely resembling the localization of GFP alone (Fig. 1A, B). To confirm this result, whole-cell extracts prepared from transgenic *Arabidopsis* constitutively expressing FLAG-tagged RGLG3 and RGLG4 (Zhang *et al.*, 2012) were separated into cytosolic and nuclear fractions. With the subcellular compartment marker proteins H3 (nuclear) and GDPase (cytoplasmic) indicating effective separation, FLAG-RGLG3 and FLAG-RGLG4 were detected in both fractions (Fig. 1C).

### RGLG3 and RGLG4 respond differently to mycotoxin FB1 treatment

**Fig. 2.**
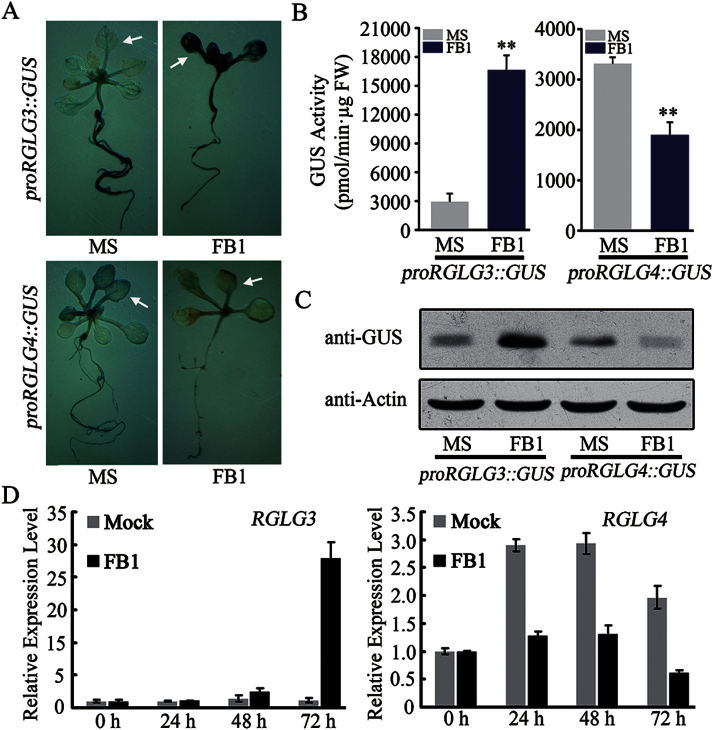
Promoter activities and transcriptional profiles of *RGLG3* and *RGLG4* after FB1 treatment. (A) Representative GUS staining of *proRGLG3::GUS* and *proRGLG4::GUS* transgenic plants after FB1 treatment. Five-day-old seedlings were treated with 2 μM FB1 for six days before GUS staining. Arrows indicate substantial changes in seedling leaves. (B) Quantified GUS activities in plants treated as in A. In each experiment, ~40 seedlings were treated and separated into four groups as four replicates for quantifying GUS activity. Bars indicate the mean **+**SD from the four replicates. Asterisks indicate a significant difference from mock (MS) treatment (Student’s t-test: *, P<0.05; **, P<0.01). (C) GUS protein levels assessed by western blot using GUS antibody in plants treated as in A. Actin was detected as a loading control. (D) Quantitative real-time PCR examination of *RGLG3* and *RGLG4* expression. RNA was extracted from 4-week-old *Arabidopsis* leaves at the indicated time points after infiltration with 10mM MgCl_2_ (mock) or 10 μM FB1 in 10mM MgCl_2_ (FB1). *UBQ10* was used as an internal control for real-time PCR, and expression levels were normalized to that measured at time point 0. Error bars indicate the SD from the mean of four technical replicates. All experiments were repeated three times with similar results.

To further explore the biological functions of RGLG3 and RGLG4 in *Arabidopsis*, transgenic plants expressing *promoter::GUS* constructs of *RGLG3* and *RGLG4* (Zhang *et al.*, 2012) were assessed for promoter activity in the presence of several chemicals. Histochemical staining, GUS activity quantification and western blotting all indicated that FB1 stimulated *RGLG3* promoter activity, whereas it suppressed that of *RGLG4* (Fig. 2A, B, C). To confirm this conclusion, transcriptional responses of *RGLG3* and *RGLG4* to FB1 were examined by real-time PCR using adult leaves that were injected with FB1. As shown in Fig. 2D, the injection method in adult leaves may cause some kind of mechanical stimulation to expression of both *RGLG3* and *RGLG4*, however, after FB1 was injected, this stimulation could be highly enhanced for *RGLG3*, but suppressed for *RGLG4* compared to the mock treatment, consistent to the results in seedlings. Even though these responses varied, they both suggest that *RGLG3* and *RGLG4* might be involved in FB1-induced programmed cell death.

### FB1-elicited PCD requires coordinated roles of RGLG3 and RGLG4

**Fig. 3.**
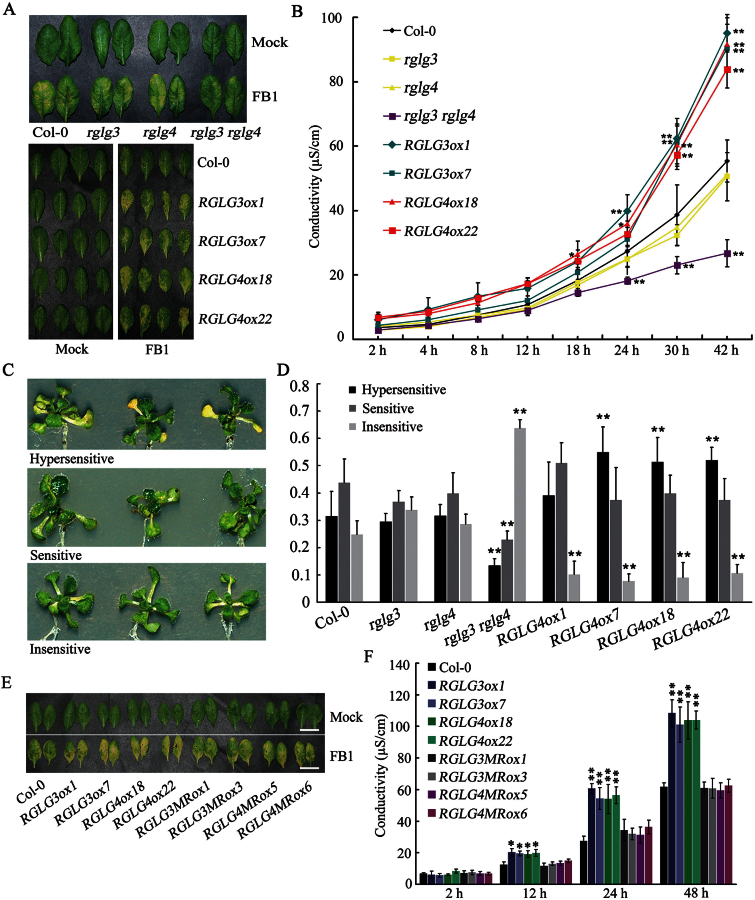
Roles of RGLG3 and RGLG4 in FB1-triggered cell death. (A) Lesion development indicating FB1-induced cell death in mutants *rglg3*, *rglg4* and *rglg3 rglg4* (upper panel) or in transgenic lines *RGLG3ox1*, *RGLG3ox7*, *RGLG4ox18* and *RGLG4ox22* (lower panel) compared with Col-0 plants. Four-week-old leaves were photographed 72h after they were infiltrated with 10mM MgCl_2_ (mock) or 10 μM FB1 in 10mM MgCl_2_ (FB1). (B) Ion leakage measurements (0–42h after sampling) in leaf disks from the plants used in A. Data indicate the mean ±SD from four replicates. Asterisks indicate a significant difference from the wild-type at the same time point (Student’s t-test: *, P<0.05; **, P<0.01). This representative experiment was repeated three times with similar results. (C, D) Altered FB1 sensitivity in mutant and transgenic seedlings of RGLG3 (C) and RGLG4 (D). One-week-old seedlings of each genotype were treated with 2 μM FB1 for six days and then classified as hypersensitive, sensitive or insensitive according to their responses to FB1 (left). The proportion of seedlings in each category is shown as the mean +SD (n>50) from three replicates. Asterisks indicate a significant difference from the wild-type (Student’s t-test: *, P<0.05; **, P<0.01). This experiment was repeated three times with similar results. (E) FB1-triggered lesion development on the leaves of transgenic *Arabidopsis* overexpressing RING domain-mutated RGLG3 or RGLG4 compared with those overexpressing wild-type RGLG3 or RGLG4. Two lines for each transgene were used, and four-week-old leaves were photographed 72h after infiltration with 10mM MgCl_2_ (mock) or 10 μM FB1 in 10mM MgCl_2_ (FB1). The experiments were repeated at least three times and yielded similar results. Bars (white), 1cm. (F) Ion leakage measurements for the plants used in E. Leaf discs were sampled for conductivity recording 72h after treatment, and the X-axis indicates the time after sampling. Data indicate the mean ±SD from four replicates. Asterisks indicate a significant difference from the wild type at the same time point (Student’s t-test: *, P<0.05; **, P<0.01). The experiments were repeated three times with similar results.

*RGLG3* and *RGLG4* mutants and transgenes (Zhang *et al.*, 2012) were then used to study whether these genes regulate FB1-induced PCD. As shown in Fig. 3A, three days after four-week-old plants were injected with 10 µM FB1 to initiate PCD, the leaves of wild-type (WT), *rglg3* and *rglg4* plants all turned yellow except for those from the double mutant *rglg3 rglg4*; in contrast, all the overexpression lines of RGLG3 and RGLG4 became more sensitive to FB1 than WT, suggesting RGLG3 and RGLG4 positively and redundantly regulate PCD elicited by FB1 treatment. Consistent with these observations, we recorded changes in ion leakage at different time points after treatment. *rglg3 rglg4* leaves underwent a less severe change in ion leakage compared with WT or either of the single mutants, whereas overexpressing either RGLG3 or RGLG4 promoted changes in ion leakage (Fig. 3B).

Similar results were observed in whole-seedling phenotypes classified according to their FB1 sensitivity (insensitive, sensitive, hypersensitive; Fig. 3C). Col-0, *rglg3* and *rglg4* showed no significant difference among the three sensitivity groups, whereas all overexpression lines had more hypersensitive and sensitive seedlings and *rglg3 rglg4* had more insensitive seedlings (Fig. 3D). Taken together, these results demonstrate that RGLG3 and RGLG4 coordinately and positively regulate FB1-induced PCD.

Both RGLG3 and RGLG4 possess ubiquitin ligase (E3) activities (Zhang *et al.*, 2012). To see if RGLG3 and RGLG4 regulate FB1-induced PCD by acting as E3s, transgenic plants overexpressing RING domain-mutated RGLG3 or RGLG4 were used for analysing cell death in comparison with unmutated RGLG3 and RGLG4. Both the visual observation of cell death in leaves and ion leakage measurements indicated that when the RING domain was mutated, RGLG3 and RGLG4 did not promote PCD (Fig. 3E, F), suggesting that E3 activities of both proteins are responsible for their functions and that they may target an unknown protein(s) for ubiquitination in regulating FB1-induced PCD.

### Transcriptional responses of RGLG3 and RGLG4 to FB1 require an intact JA pathway

**Fig. 4.**
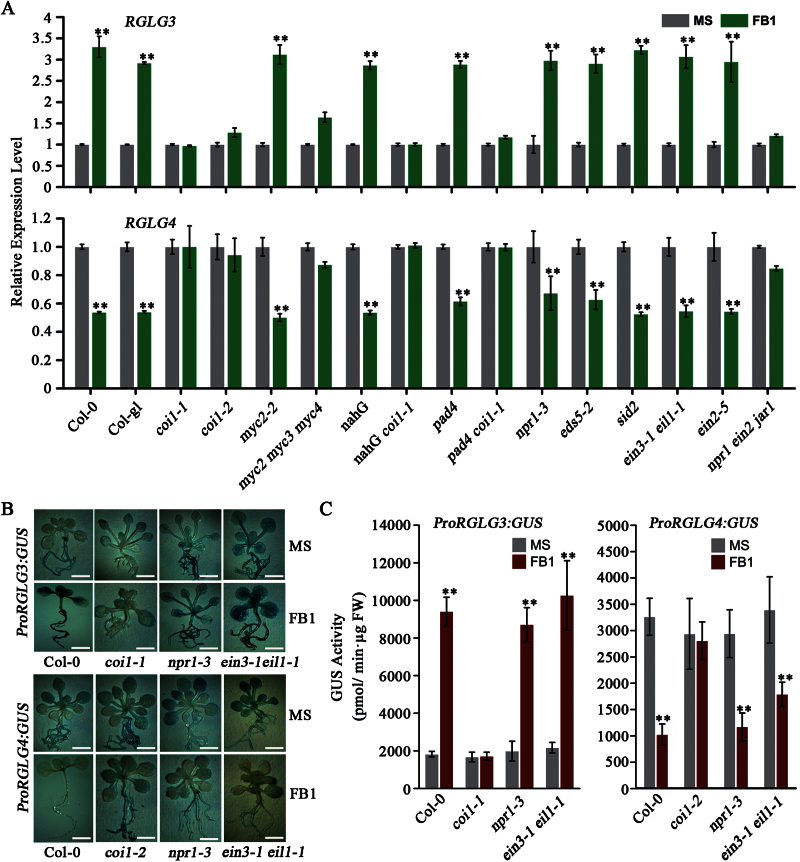
JA pathway-dependent FB1 responsiveness of *RGLG3* and *RGLG4*. (A) FB1-elicited expression changes of *RGLG3* and *RGLG4* in Col-0, *coi1*, *myc2-2*, *myc2 myc3 myc4*, *NahG*, *NahG coi1-1*, *pad4*, *pad4 coi1-1*, *npr1-3*, *eds5-1*, *ein3-1 eil1-1*, *ein2-5* and *npr1-1 ein2-1 jar1-1* plants. Five-day-old seedlings were treated with or without 2 μM FB1 and grown for six days. Expression levels were determined by real-time PCR. *UBQ10* was used as an internal control, and expression in mock-treated plants was considered as 1.0. Bars indicate the mean ±SD from four technical replicates. Asterisks indicate a significant difference from mock (MS) treatment (Student’s t-test: *, P<0.05; **, P<0.01). (B) Representative GUS staining of transgenic plants in Col-0, *coi1*, *npr1-3* and *ein3-1 eil1-1* backgrounds expressing *proRGLG3::GUS* or *proRGLG4::GUS*. Seedlings were grown and treated as in A. Bars, 5mm. (C) Quantified GUS activity from plants as in B. In each experiment, ~30 seedlings were treated and separated into three groups as three replicates. Bars indicate the mean ±SD of these three replicates. Asterisks indicate a significant difference from mock (MS) treatment (Student’s t-test: *, P<0.05; **, P<0.01). All experiments were repeated three times with similar results.

The JA, SA and ET signalling pathways are required for FB1-induced PCD (Asai *et al.*, 2000), and they may be involved in RGLG3 and RGLG4 functions. We used hormone pathway mutants to determine which hormone pathway(s) controls the responsiveness of *RGLG3* and *RGLG4* to FB1. The following mutants were assessed: *coi1-1* (Xie *et al.*, 1998), *coi1-2* (Xu *et al.*, 2002), *myc2-2* (Boter *et al.*, 2004) and *myc2 myc3 myc4* (Fernandez-Calvo *et al.*, 2011) for JA; *pad4* (Jirage *et al.*, 1999)*, eds5-1* (Glazebrook *et al.*, 1996), *sid2* (Wildermuth *et al.*, 2001), *npr1-3* (Cao *et al.*, 1994) and the transgenic line *NahG* (van Wees and Glazebrook, 2003) for SA and *ein2-5* (Guzman and Ecker, 1990) and *eil1-1 ein3-1* (Alonso *et al.*, 2003) for ET. In addition, the double or triple crosses *pad4 coi1-1*, *NahG coi1-1* and *npr1-1 ein2-1 jar1-1* (Clarke *et al.*, 2000), which disrupt two or three pathways, respectively, were analysed. Plants were treated with 2 µM FB1, and *RGLG3* and *RGLG4* expression levels were quantified. FB1 enhanced *RGLG3* expression and suppressed *RGLG4* expression in all genotypes except for mutants with severe JA pathway defects, including *coi1, myc2 myc3 myc4* and *npr1-1 ein2-1 jar1-1* (Fig. 4A). *proRGLG3::GUS* and *proRGLG4::GUS* reporter constructs introduced into the Col-0, *coi1*, *npr1-3* and *ein3-1 eil1-1* genetic backgrounds were then used to determine *RGLG3* and *RGLG4* promoter activities upon FB1 treatment. Both histochemical staining and quantification of enzymatic activity showed that FB1 caused obvious alteration in all the backgrounds but *coi1* (Fig. 4B, C), consistent with the transcript quantification results. Together, these expression analyses indicate that the JA pathway is required for RGLG3 and RGLG4 responses to FB1.

### Roles of RGLG3 and RGLG4 in FB1-induced cell death require an intact JA signalling pathway

**Fig. 5.**
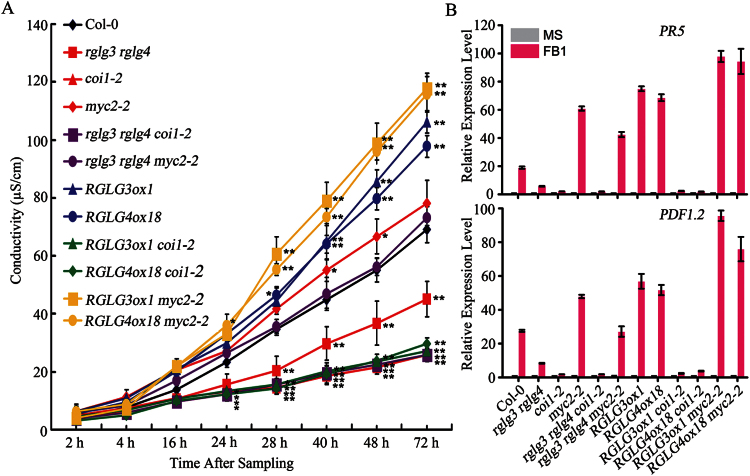
Roles of the JA pathway in RGLG3- and RGLG4-mediated cell death. (A) Conductivity measurements in Col-0, *rglg3 rglg4*, *coi1-2*, *myc2-2*, *rglg3 rglg4 coi1-2*, *rglg3 rglg4 myc2-2*, *RGLG3ox1*, *RGLG4ox18*, *RGLG3ox1 coi1-2*, *RGLG4ox18 coi1-2*, *RGLG3ox1 myc2-2* and *RGLG4ox18 myc2-2* after FB1 treatment. Treatment and ion leakage quantification were as in Fig. 3B; bars show the mean ±SD from three independent replicates at different time points after sampling. Asterisks indicate a significant difference from the wild-type at the same time point (Student’s t-test: *, P<0.05; **, P<0.01). The experiments were repeated three times with similar results. (B) FB1-induced expression of *PDF1.2* and *PR5* in genotypes used in A. Five-day-old seedlings were treated as in Fig. 2A, and transcription levels were examined by real-time PCR. *UBQ10* was used as an internal control, and expression levels were normalized to that of the mock treatment. Data represent the mean ±SD from four technical replicates. The experiments were repeated three times and had similar results.

To determine if the JA signalling pathway is critical for RGLG3 and RGLG4 functions in PCD, *rglg3 rglg4*, *RGLG3ox1* and *RGLG4ox18* plants, as well as those plants crossed with either *coi1 or myc2* plants (*rglg3 rglg4 coi1-2*, *rglg3 rglg4 myc2-2*, *RGLG3ox1 coi1-2*, *RGLG4ox18 coi1-2*, *RGLG3ox1 myc2-2* and *RGLG4ox18 myc2-2)* (Zhang *et al.*, 2012) were used to investigate FB1-elicited PCD. Consistent with the finding that JA positively mediates FB1-induced cell death (Asai *et al.*, 2000), visual lesion development in *coi1-*2 leaves was less severe than in Col-0 (Supplementary Fig. S1), and the increase in ion leakage rate was also lower in *coi1-2* than in Col-0 after FB1 treatment (Fig. 5A). *coi1-2* had a lower ion leakage rate and thus greater PCD suppression than *rglg3 rglg4*, but *rglg3 rglg4 coi1-2* had similar conductivity to *coi1-2* (Fig. 5A). Thus, although enhanced RGLG3 and RGLG4 expression promoted FB1-elicited ion leakage, these effects disappeared in the *coi1-2* background (Fig. 5A). Similarly, promoted lesion development by RGLG3 or RGLG4 overexpression was repressed by *coi1-2* mutation (Supplementary Fig. S1), suggesting a determinative role for COI1 in the positive effects of RGLG3 and RGLG4 on PCD. In *myc2-2*, lesion development was more severe and conductivity increased faster than in Col-0 (Supplementary Fig. S1 and Fig. 5A), implying a negative role for MYC2 in FB1-triggered cell death, which is similar to its role in pathogen defence responses (Lorenzo *et al.*, 2004). Moreover, the attenuated ion leakage of *rglg3 rglg4* was reversed and the increased ion leakage in the overexpression lines was further facilitated by *myc2-2* (Fig. 5A), suggesting MYC2 involvement in RGLG3 and RGLG4 promotion of FB1-triggered cell death. Similar conclusion can also be drawn from lesion phenotypes (Supplementary Fig. S1).

FB1 stimulates expression of JA-responsive marker genes such as the *PLANT DEFENSIN1.2* (*PDF1.2)* and *PATHOGENESIS-RELATED5* (*PR5)* (Stone *et al.*, 2000). In *rglg3 rglg4*, FB1-induced *PDF1.2* and *PR5* expression was attenuated, but in *RGLG3ox1* and *RGLG4ox18*, their expression was enhanced compared with that in Col-0 (Fig. 5B), indicating that RGLG3 and RGLG4 can promote FB1-stimulated expression of JA marker genes. FB1-induced *PDF1.2* and *PR5* expression in the JA-insensitive *coi1-2*, *rglg3 rglg4 coi1-2*, *RGLG3ox1 coi1-2* and *RGLG4ox18 coi1-2* was similarly more down-regulated than in *rglg3 rglg4* (Fig. 5B), suggesting that RGLG3 and RGLG4 effects on the FB1 responsiveness of *PDF1.2* and *PR5* were dependent on COI1. In *myc2-2*, *PDF1.2* and *PR5* expression was elevated compared with that in Col-0 (Fig. 5B), further confirming a negative role for MYC2 in FB1-triggered JA signalling. This is consistent with reports that MYC2 negatively regulates pathogen defence-related genes (Boter *et al.*, 2004; Lorenzo *et al.*, 2004). The suppressed induction of *PDF1.2* and *PR5* in *rglg3 rglg4* could be reversed in *rglg3 rglg4 myc2-2* to a level that was higher than that in Col-0 and was further enhanced in *RGLG3ox1 myc2-2* and *RGLG4ox18 myc2-2* (Fig. 5B), implying that MYC2 might act downstream of RGLG3 and RGLG4 in regulating these two FB1-responsive genes. Collectively, these data indicate that an intact JA pathway was important for RGLG3 and RGLG4 to perform their positive roles in FB1-induced PCD.

### RGLG3 and RGLG4 control FB1 responses mainly by modulating the JA pathway versus the SA pathway

**Fig. 6.**
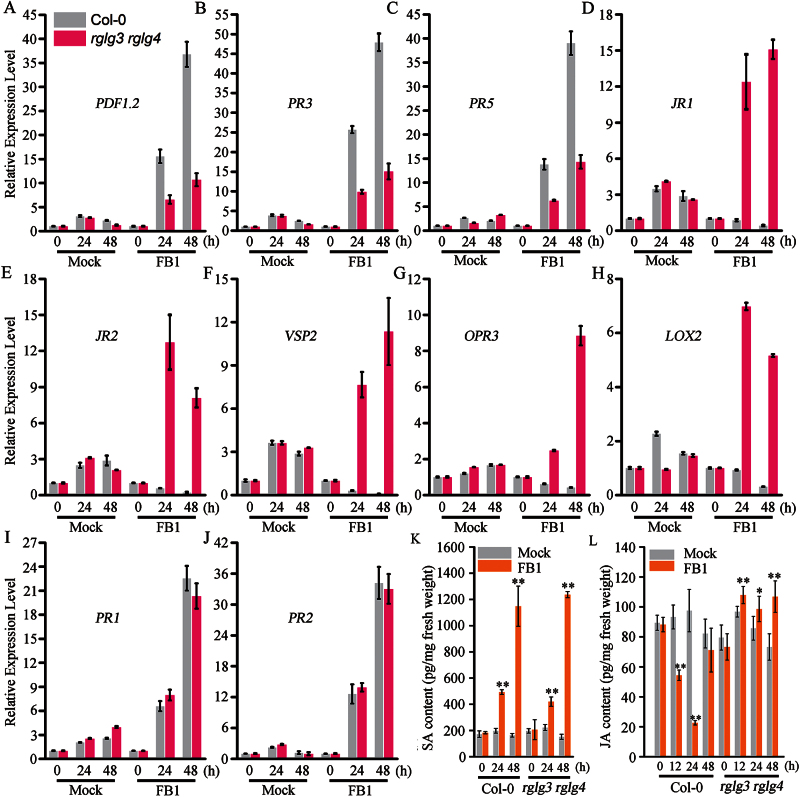
Specific roles of RGLG3 and RGLG4 in the FB1-activated JA pathway. (A–J) Expression profiles of JA- and SA-responsive genes after FB1 treatment in Col-0 and *rglg3 rglg4*. RNA was extracted from four-week-old *Arabidopsis* leaves at the indicated time points after infiltration with 10mM MgCl_2_ (mock) or 10 μM FB1 in 10mM MgCl_2_ (FB1). *UBQ10* was used as an internal control, and expression levels were normalized to that measured at time 0. Data show the mean ±SD from four technical replicates. (K, L) JA and SA quantification after FB1 treatment in Col-0 and *rglg3 rglg4*. Four-week-old *Arabidopsis* leaves were treated as in A, and then the samples were collected at the indicated times points for JA and SA measurement. Data represent the mean ±SD of three technical replicates. Asterisks indicate a significant difference from time point 0 (Student’s t-test: *, P<0.05; **, P<0.01).

As FB1 activates both JA and SA signalling in eliciting PCD (Stone *et al.*, 2000), we further examined the FB1 responsiveness of these two pathway genes in *rglg3 rglg4* and Col-0 using mature *Arabidopsis* leaves. FB1 treatment induced expression of the defence-related genes, such as *PR3*, *PR5* and *PDF1.2* (Stone *et al.*, 2000) compared to the mock treatment (Fig. 6A, B, C), and similar to the assays in young seedlings (Fig. 5B), FB1 induction was suppressed in *rglg3 rglg4* compared with wild type (Fig. 6A, B, C). However, additional JA-responsive genes that are related to growth repression (Lorenzo *et al.*, 2004), including *JASMONIC ACID RESPONSIVE1* (*JR1)* (Rojo *et al.*, 1999)*, JR2* and *VEGETATIVE STORAGE PROTEIN2* (*VSP2*) (Staswick *et al.*, 1991), and to JA biosynthesis, such as *12-OXOPHYTODIENOATE REDUCTASE3* (*OPR3)* (Stintzi and Browse, 2000) and *LIPOXYGENASE2 (LOX2)* (Bell and Mullet, 1993), were obviously suppressed by FB1 in Col-0 compared to the mock treatment, and this effect disappeared in *rglg3 rglg4* plants, in which these genes even became inducible in response to FB1 (Fig. 6D, E, F, G, H). In contrast, FB1 induction of the typical SA-responsive genes *PR1* and *PR2* (Stone *et al.*, 2000) was not apparently affected in *rglg3 rglg4* versus Col-0 (Fig. 6I, J). Consistent with these expression profiles, hormone quantification showed FB1 indeed inhibited total JA production after FB1 infiltration in Col-0 compared to the mock treatment and this suppression was counteracted in *rglg3 rglg4* (Fig. 6L). FB1-induced SA biosynthesis was not apparently affected in *rglg3 rglg4* in comparison to that in Col-0 (Fig. 6K). Therefore, RGLG3 and RGLG4 mainly modulate the JA pathway during FB1 treatment.

### RGLG3 and RGLG4 mediate SA suppression of JA signalling in FB1-induced responses

**Fig. 7.**
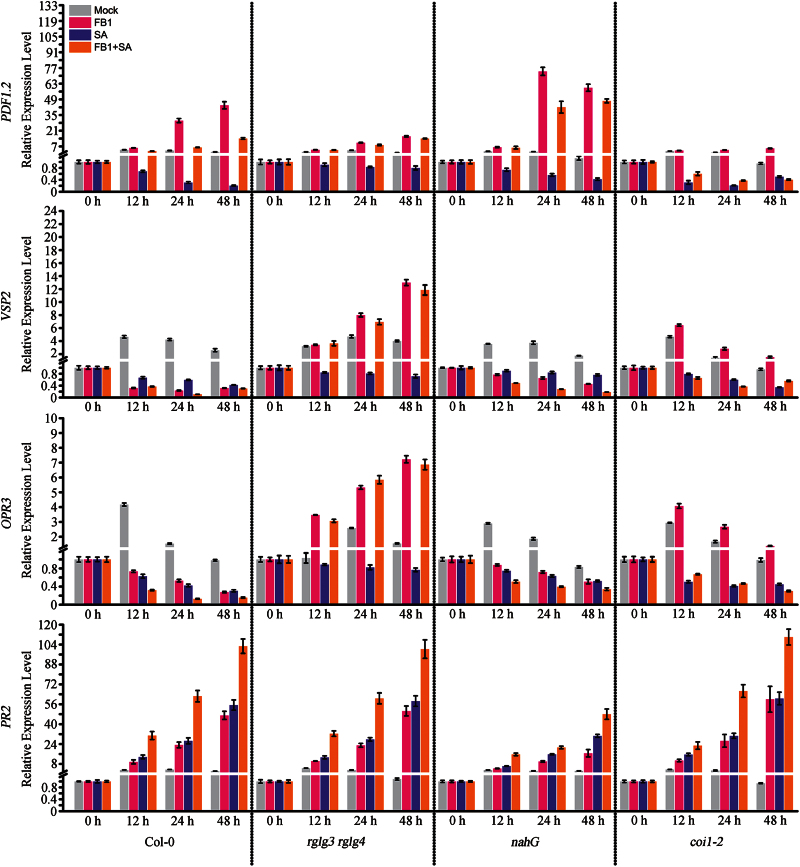
Roles of RGLG3 and RGLG4 in SA suppression of the JA pathway. Four-week-old *Arabidopsis* leaves of the indicated genetic backgrounds were infiltrated with 10mM MgCl_2_ (mock) or 10 μM FB1 in 10mM MgCl_2_ (FB1), were sprayed directly with 2mM SA (SA) or were given a combined FB1 and SA treatment (FB1+SA). Expression of *PDF1.2*, *VSP2*, *OPR3* and *PR2* was assessed by real-time PCR. *UBQ10* was used as an internal control, and expression levels were normalized to that measured at time 0. Data indicate the mean ±SD from four technical replicates. This experiment was repeated three times with similar results.

Crosstalk between SA and JA pathways has been widely documented in diverse pathogen defence responses (Pieterse *et al.*, 2012), but it remains uncharacterized in the FB1 system. We examined how SA affected JA signalling after FB1 treatment and determined if RGLG3 and RGLG4 were involved. In Col-0, both exogenous SA application and FB1 injection promoted SA-responsive *PR2* expression, and this induction was elevated by combined SA and FB1 treatment; in the SA-deficient *NahG* transgenic plant, *PR2* induction by SA, FB1 or a combined treatment was attenuated; in both *coi1-2* and *rglg3 rglg4, PR2* induction by the three treatments was not substantially affected (Fig. 7). SA application suppressed all the checked JA-responsive genes in Col-0, consistent with previous reports (Leon-Reyes *et al.*, 2010; Van der Does *et al.*, 2013). This response was, however, different from that following FB1 treatment, which stimulated defence-related *PDF1.2* while repressing growth-related *VSP2* and biosynthesis-related *OPR3.* When SA and FB1 treatments were combined, *PDF1.2* was only moderately inducible, and *OPR3* and *VSP2* were more severely inhibited in Col-0 (Fig. 7). Interestingly, SA repression was similarly reduced in *rglg3 rglg4* and *NahG*, but remained unchanged in *coi1-2*. Moreover, the FB1 effect on *PDF1.2* expression was promoted by the *NahG* transgene, whereas it was blocked in *coi1-2* and *rglg3 rglg4* plants; FB1 repression of *OPR3* and *VSP2* decreased in *NahG* plants, but in *rglg3 rglg4* and *coi1-2* plants these genes were induced (Fig. 7). The combined treatment with SA and FB1 in *NahG* mitigated FB1 induction of *PDF1.2,* whereas it promoted repression of *OPR3* and *VSP2*, although not as severely as in Col-0 (Fig. 7). Altogether, these data indicate that RGLG3 and RGLG4 also mediate SA suppression of the JA pathway upon FB1 treatment.

## Discussion

The mycotoxin FB1 provides a useful pathogen-free system for studying PCD during the defence response (Stone *et al.*, 2000). Our characterization of RGLG3 and RGLG4 in this process has underscored the contribution of the JA pathway to FB1-induced PCD.

Hormone pathways, including SA, JA and ET, have important roles in FB1-induced PCD (Asai *et al.*, 2000), although the actual roles of these hormone pathways have not been determined in plants. Previous work using JA-resistant *jar1* protoplast has indicated a positive role for the JA pathway in FB1-induced cell death (Asai *et al.*, 2000). Here we show in adult plants that the JA receptor mutant *coi1-2* was insensitive to FB1 (Fig. 5A), and FB1-induced *PDF1.2* and *PR5* expression was also suppressed in *coi1-2* (Fig. 5B). Therefore, JA perception is required for FB1-triggered responses. In addition, similar to the effect in JA pathway-mediated defence responses (Dombrecht *et al.*, 2007), mutating the essential regulator MYC2 facilitated FB1-induced cell death (Fig. 5A) and promoted FB1 induction of *PDF1.2* and *PR5* expression (Fig. 5B), suggesting a negative role for MYC2 in this process. Furthermore, both genetic evidence and molecular evidence provided here indicate that RGLG3 and RGLG4, two recently characterized upstream regulators of the JA pathway (Zhang *et al.*, 2012), also coordinately regulate FB1-triggered responses in a COI1- and MYC2-dependent manner (Fig. 5). Their importance in FB1-induced PCD can be further supported by the expression profiling of lesion mimic genes (Supplementary Fig. S2), mutants of which display spontaneous PCD-like lesions, thus providing useful tools to study PCD pathways in plants (Lorrain *et al.*, 2003). Consistently, *RGLG3* and *RGLG4* expression was similarly affected by FB1 and JA [Fig. 5 and Zhang *et al.* (2012)], suggesting that the mechanism used by JA to tightly regulate the protein levels of these genes also functions in the FB1-triggered response. Taken together, these findings strongly suggest that the JA pathway promotes FB1-induced PCD.

The JA pathway also promotes parasitism by the hemibiotrophic pathogen *Pseudomonas syringae* pv. *tomato* DC3000 (*Pst* DC3000) (Thomma *et al.*, 1998), whereas RGLG3 and RGLG4 support *Pst* DC3000 growth in infected *Arabidopsis* leaves (Zhang *et al.*, 2012). These observations are contradictory to the dominant roles of JA in defence responses against necropathogenic pathogens, such as *Alternaria brassicicola* and *Botrytis cinerea* (Pieterse *et al.*, 2009), and the molecular basis for these differences remains unclear. Interestingly, *FB1-resistant 1* (*fbr1*) and *fbr2* are insensitive to FB1 but also resistant to *Pst* DC3000 (Stone *et al.*, 2000). Consideration of possible roles of the JA pathway may help to elucidate the underlying mechanism behind these effects. Two other FB1-resistant mutants, *fbr11* (Teng *et al.*, 2008) and *fbr12* (Feng *et al.*, 2007), have defects in pollen development and sterility, characteristics that are probably related to JA deficiency but have not been verified.

However, it is also possible that FB1 inhibited JA production (Fig. 6), probably resulting from feedback control, as JA biosynthesis undergoes positive feedback regulation (Turner *et al.*, 2002), but growth-related genes and JA biosynthesis genes were suppressed by FB1 (Figs 6, 7). This suggests that new JA production (or high-level JA production) is disadvantageous for infection by FBI-producing pathogens. How FB1 can activate JA signalling is not clear. There may be a preexisting pool of inactive JA (Fonseca *et al.*, 2009), and FB1 may stimulate this low-level pool and activate JA perception and downstream signalling. By this means, these pathogens may hijack the JA pathway to initiate cell death in favour of their invasion, meanwhile preventing host plants from producing high-level JA for host defence. A similar JA pathway-hijacking strategy is used by *Pst* DC3000, which secretes the JA-Ile mimic coronatine to activate JA signalling during the invasion process (Fonseca *et al.*, 2009).

Another way to repress JA production by FB1 may come from SA suppression of JA signalling. Crosstalk between SA and JA pathways has been considered a mechanism for prioritizing limited resources for better defence according to the context of different stresses (Pieterse *et al.*, 2012). FB1 activated both pathways, as disrupting either one in *NahG* or *coi1-2* plants significantly attenuated the FB1-induced transcriptome (Fig. 7). Moreover, exogenous SA inhibited JA-responsive genes; FB1 induction of *PDF1.2* was enhanced, and FB1 suppression of *VSP2* and *OPR3* was mitigated in *NahG* plants (Fig. 7). These results imply that activated SA signalling promotes FB1-suppressed JA production, which may further contribute to the defence response of the host plant upon FB1 injection. In contrast, *Pst* DC3000-activated JA signalling is accompanied by suppression of the SA pathway (Kloek *et al.*, 2001).

RGLG3 and RGLG4 emerge as novel players mediating SA-JA crosstalk based on our results (Fig. 7). However, in the JA pathway RGLG3 and RGLG4 function upstream of COI1 (Zhang *et al.*, 2012), and SA suppresses JA signalling downstream of COI1 (Van der Does *et al.*, 2013). Therefore, RGLG3 and RGLG4 probably have another downstream target(s). Notably, RGLG3 and RGLG4 are different from previously identified factors, such as NPR1 (Spoel *et al.*, 2003), GRX48 (Ndamukong *et al.*, 2007), WRKY70 (Li *et al.*, 2004) and MPK4 (Petersen *et al.*, 2000), in that they have no effect on FB1-triggered SA production or signalling (Fig. 6) and the SA pathway is not required for their transcriptional regulation (Fig. 4). So far, most evidence indicates that SA repression targets JA transcriptional machinery (Pieterse *et al.*, 2012; Van der Does *et al.*, 2013). In addition, RGLG3- and RGLG4-modulated SA inhibition suggests that SA interferes with the turnover of certain transcription regulator(s) in the JA pathway.

Our data, together with previous reports, indicate that the four RGLGs in *Arabidopsis* have different spatial distributions in the cell, with RGLG1 and RGLG2 localized to the plasma membrane (Stone *et al.*, 2005; Yin *et al.*, 2007), whereas RGLG3 and RGLG4 are located in the plasma and nucleus (Fig. 1). The second glycine residue is important for the membrane location of RGLG2 (Yin *et al.*, 2007), but RGLG3 and RGLG4 totally lack the N-terminal sequence that is present in RGLG1 and RGLG2 (Zhang *et al.*, 2012), so the N-terminal differences in RGLG3 and RGLG4 probably account for their distinct localizations. A recent report indicates that RGLG2 translocates into the nucleus upon stress (Cheng *et al.*, 2012). We did use exogenous FB1 to treat roots from transgenic *Arabidopsis* plants in our study, but no notable changes in RGLG3 and RGLG4 localization were observed (unpublished data), which suggests that they have different modes of action in the cell relative to RGLG1 and RGLG2. Protein ubiquitination has important roles in regulating cellular responses (Vierstra, 2009). For example, a membrane-localized Ring-type E3, RING1, negatively regulates FB1-triggered cell death (Lin *et al.*, 2008), and other E3s, such as DAl1 and DAl2 (Basnayake *et al.*, 2011), are also involved in FB1-triggered cell death. Therefore, the induction of PCD by FB1 is a complicated process involving multiple E3s with different roles at different locations in the cell. Substantially more work will be required to characterize the RGLG targets and other factors to fully appreciate the nature of FB1-induced cell death.

In summary, our data highlight the significance of the JA pathway in FB1-induced PCD and reveal the essential roles of two ubiquitin ligases, RGLG3 and RGLG4, in mediating this process. RGLG3 and RGLG4 may act as a regulatory cluster in the complex hormone network controlling cell death (Supplementary Fig. S3), and thus identifying their target(s) will no doubt further our understanding of the mechanisms of FB1-elicited PCD as well as JA signalling.

## Supplementary data

Supplementary data are available at JXB online.

Supplementary Fig. S1. Representative leaves showing lesion development after FB1 treatment.

Supplementary Fig. S2. FB1 responsiveness of lesion mimic mutant (LMM) genes in *rglg3 rglg4* plants versus Col-0 plants.

Supplementary Fig. S3. A model illustrating possible roles of RGLG3 and RGLG4 in FB1-triggered cell death.

Supplementary Table S1. List of primers used in this study.

Supplementary Data
