# Supplementary material for: Hijacking of the jasmonate pathway by the mycotoxin fumonisin B1 (FB1) to initiate programmed cell death in Arabidopsis is modulated by RGLG3 and RGLG4
Source: J Exp Bot. 2015 Mar 18;66(9):2709–21. doi: 10.1093/jxb/erv068 (PMC4986873; doi:10.1093/jxb/erv068)
Supplement: Supplementary Data [file supp_66_9_2709__index.html]

Hijacking of the jasmonate pathway by the mycotoxin fumonisin B1 (FB1) to initiate programmed cell death in Arabidopsis is modulated by RGLG3 and RGLG4 — Hijacking of the jasmonate pathway by the mycotoxin fumonisin B1 (FB1) to initiate programmed cell death in Arabidopsis is modulated by RGLG3 and RGLG4 — Supplementary Data 

# Hijacking of the jasmonate pathway by the mycotoxin fumonisin B1 (FB1) to initiate programmed cell death in *Arabidopsis* is modulated by RGLG3 and RGLG4

## Supplementary Data

Data files

**Files in this Data Supplement:**

- Supplementary Data - Supplementary Data
