# Supplementary material for: Hijacking of the jasmonate pathway by the mycotoxin fumonisin B1 (FB1) to initiate programmed cell death in Arabidopsis is modulated by RGLG3 and RGLG4
Source: J Exp Bot. 2015 Mar 18;66(9):2709–21. doi: 10.1093/jxb/erv068 (PMC4986873; doi:10.1093/jxb/erv068)
Supplement: Supplementary Data [file supp_erv068_jexbot135814_file001.pdf]

Journal of Experimental Botany

The mycotoxin fumonisin B1 (FB1) hijacking of jasmonate pathway to initiate programmed cell death in Arabidopsis is modulated by RGLG3 and RGLG4

Xu Zhang, Qian Wu, Shao Cui, Jiao Ren, Wanqiang Qian, Yang Yang, Shanping He , Jinfang Chu, Xiaohong Sun, Cunyu Yan, Xiangchun Yu and Chengcai An

Supplementary Data

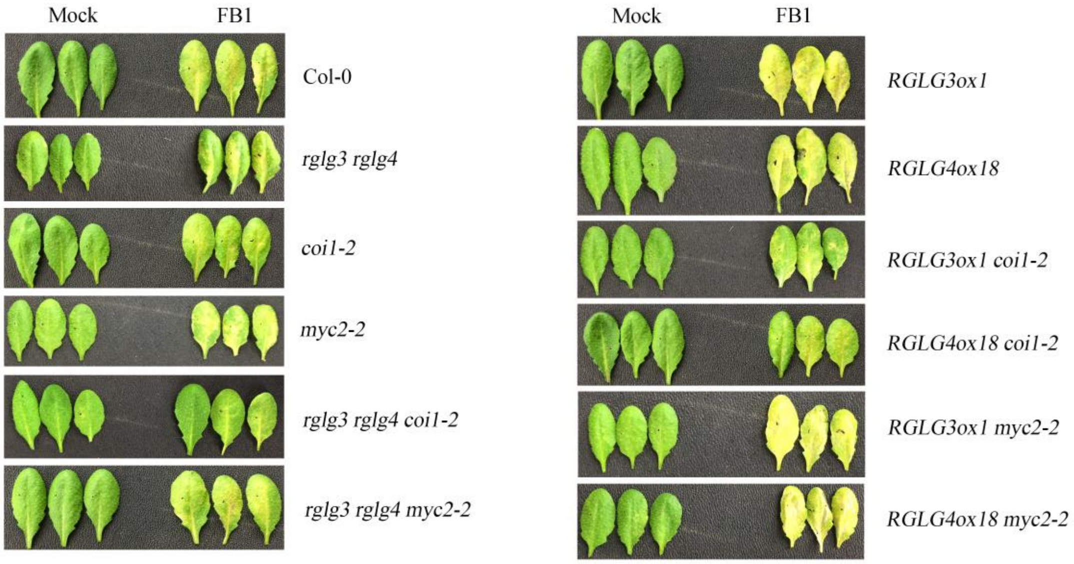

Figure S1. Representative leaves showing lesion development after FB1 treatment. Four-week-old leaves of Col-0, *rglg3 rglg4*, *coi1-2*, *myc2-2*, *rglg3 rglg4 coi1-2*, *rglg3 rglg4 myc2-2*, *RGLG3ox1*, *RGLG4ox18*, *RGLG3ox1 coi1-2*, *RGLG4ox18 coi1-2*, *RGLG3ox1 myc2-2*, and *RGLG4ox18 myc2-2* were photographed 72 h after infiltration with 10 mM $MgCl_2$ (Mock) or 10 μM FB1 supplied in 10 mM $MgCl_2$ (FB1).

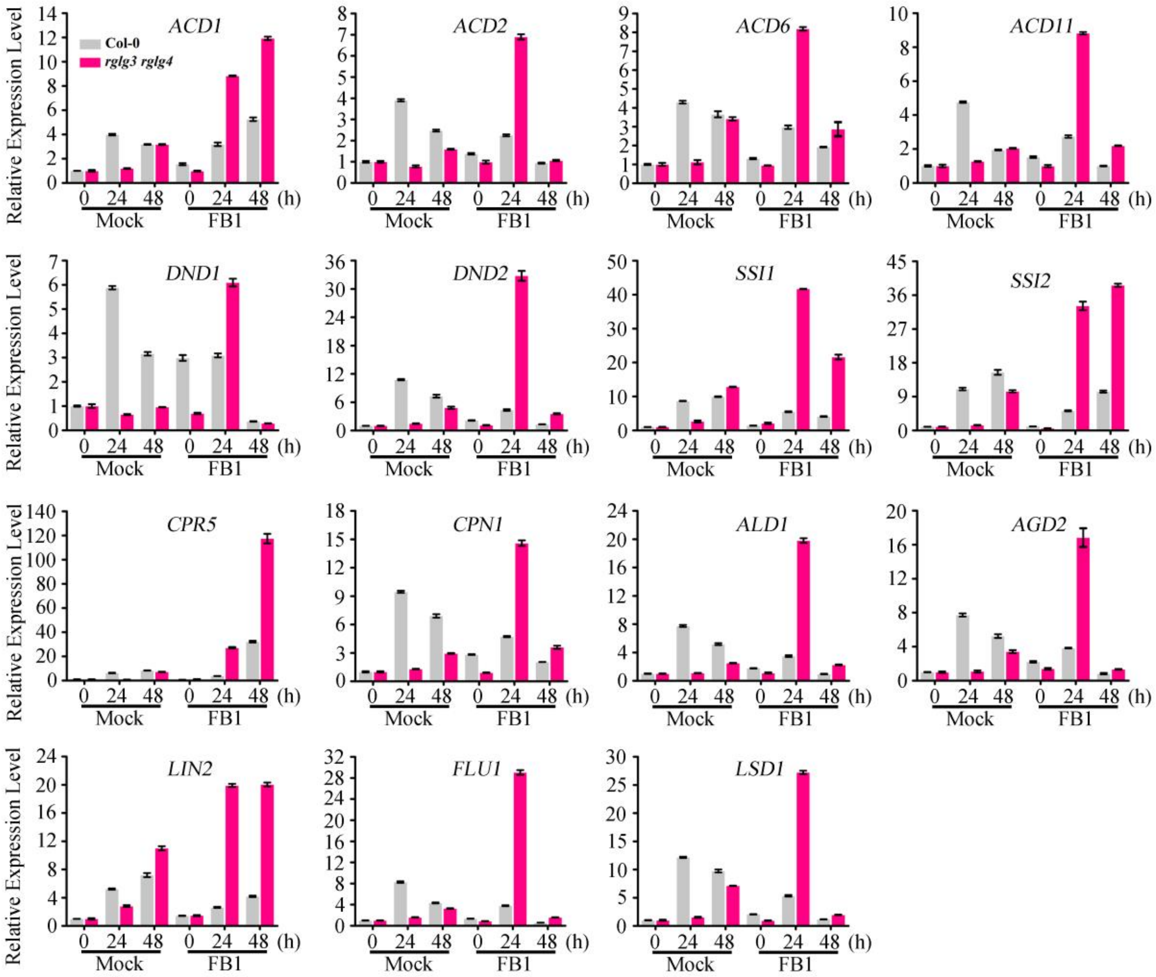

Figure S2. FB1 responsiveness of lesion mimic mutant (LMM) genes in *rglg3 rglg4* versus Col-0. Expression of LMM genes after FB1 treatment was determined by realtime PCR. 4-week-old Arabidopsis leaves were infiltrated with 10 mM $MgCl_2$ (Mock) or 10 μM FB1 in 10 mM $MgCl_2$ (FB1). Then the samples were collected at the indicated time points for RNA extraction. *UBQ10* was used as an internal control, and expression levels were normalized to that measured at time point 0. Error bars indicate ±SD from the means of three technical replicates. This experiment was repeated three times with similar results.

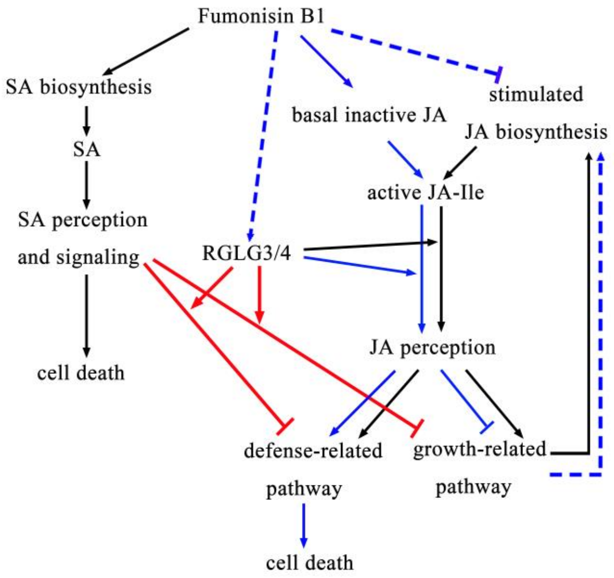

Figure S3. A model illustrating possible roles of RGLG3 and RGLG4 in FB1-triggered cell death. Invasion by pathogens that produce Fumonisin B1 activates both SA and JA pathways in Arabidopsis. On one side, FB1 probably activates low-level pre-exising inactive JA, thus highjacking the well-established JA signaling pathway to initiate cell death, but concomitantly inhibiting newly (high-level) JA biosynthesis directly or by feedback regulation through the JA growth repression-related pathway, all for the good of pathogen growth. In this process, RGLG3 and RGLG4 probably act as regulators upstream of JA receptor component COI1. On the other side, FB1 activates plant defense system and the SA pathway is activated, as a final result, cell death takes places as a mechanism to defend the pathogen invasion. During this process, SA further suppresses JA signaling for effective defense, which is also modulated by RGLG3 and RGLG4, probably by targeting a downstream regulator of JA pathway. The black lines indicate well-known SA and JA signaling pathway, the blue lines indicate FB1-activated signaling that use JA pathway to initiate cell death, while the red lines indicate RGLG3 and RGLG4-modulated SA-suppression of JA pathway, and the dotted lines indicate possible regulation but not addressed by our data.

Table S1. List of primers used in this study.

Name	Sequence	Function
RGLG3-S- BamH Ⅰ	5'-GGGGGGATCCATGAACATGAGCAATAAACGTAATCA-3'	pRTL-GFP-RGLG3
RGLG3-A-Spe Ⅰ	5'-GGGACTAGTTCAAGTGTACAGCCTTATCCTTG-3'	
RGLG4-S-Bgl Ⅱ	5’-GCGCAGATCTATGACTATGGGGAATTTCTTAA-3’	pRTL-GFP-RGLG4
RGLG4-A-Xba Ⅰ	5’-GCGCTCTAGATCATGTGTAAAGCTTTAGCCG-3’	
RGLG3-RS	5'- AGAGCACAAAGATGCGGCTAAGA-3'	Real-Time PCR
RGLG3-RA	5'-TCACGCTCTATAACCTCTGGTGGT-3'	
RGLG4-RS	5'-ATCCCTAAGCGTGAATTCGACAAC-3'	Real-Time PCR
RGLG4-RA	5'-GTTCGATGGCTGCTTGATACTGA-3'	
ACD1-RS	5'-GTTCTAGCATTCAGAAACTGGCTCA-3'	Real-Time PCR
ACD1-RA	5'-CTCCTTTGCAGGAAGAGCATACTTG-3'	
ACD2-RS	5'-GAGCAGTTCCAAGTCGCTTGTG-3'	Real-Time PCR
ACD2-RA	5'-GTCGATGAGAATCAAGAGCAGTGTC-3'	
ACD6-RS	5'-ACAGTCAGTGCCACTAGACCCTAAG-3'	Real-Time PCR
ACD6-RA	5'-AGTTTGGTTTTTTGCTATCGCTG-3'	
ACD11-RS	5'-GAACAGATCATAGCTTCCGAAGG-3'	Real-Time PCR
ACD11-RA	5'-TGGGAAGAGCATACATCCCAAG-3'	
DND1-RS	5'-GCTTTGATCTCATTAACAAGGTGC-3'	Real-Time PCR
DND1-RA	5'-GTACAGGATCTCCTTCACGGATGA-3'	
DND2-RS	5'-AGGCTACCGCCTTCTTCTTCAAC-3'	Real-Time PCR
DND2-RA	5'-CGAGCACTACGTTTGACTTTCTCG-3'	
SSI1-RS	5'-TCAATGTCCCCATCTCTCATCG-3'	Real-Time PCR
SSI1-RA	5'-CCTCCAGTGCCATGAACAACTG-3'	
SSI2-RS	5'-TTCATCTCTCACGGAAACACAGC-3'	Real-Time PCR
SSI2-RA	5'-AAGCCATGACAGTACCATCAGGA-3'	
CPR5-RS	5'-CGATCATCAGGTACGAAGCAGAC-3'	Real-Time PCR
CPR5-RA	5'-ACTATTAGCCAGAGTTTGCCATCAC-3'	
CPN1-RS	5'-GCGTCGCTAGCTCAAGGTTCAC-3'	Real-Time PCR
CPN1-RA	5'-GATTGATAAGGGCAAATCCGATG-3'	
ALD1-RS	5'-CCACTTCCTTCAACGGAGCTTC-3'	Real-Time PCR
ALD1-RA	5'-CAAGCGAGACTAGAGTGTCCATCAG-3'	
AGD2-RS	5'-TGGAGTTCGACTTGGTTGGACTG-3'	Real-Time PCR
AGD2-RA	5'-GCCTCAAGTCCTTCGGGTGTAAG-3'	
LIN2-RS	5'-CGAGACAGATGCTCCAAAGGATG-3'	Real-Time PCR
LIN2-RA	5'-GGTCAAATTTGTCACAGGCTTGC-3'	

FLU1-RS	5'-GCTCTTATTGGAGCCACCGTTG-3'	Real-Time PCR
FLU1-RA	5'-TCCCACTGGAGCATAACTCAAGC-3'	
LSD1-RS	5'-CATTGTCGGACGACCCTCATG-3'	Real-Time PCR
LSD1-RA	5'-CTGTTCCATTTGGCCGGTTAG-3'	
OPR3-RS	5'-GAAGGTGTAGTTTCAGCCATAGGAG-3'	Real-Time PCR
OPR3-RA	5'-AGCTTTGAGCCATTAACACCTTG-3'	
PDF1.2-RS	5'-GGCAATGGTGGAAGCACAGAAG-3'	Real-Time PCR
PDF1.2-RA	5'-TGTGTGCTGGGAAGACATAGTTGC-3'	
VSP2-RS	5'-CCCATCATACTCAGTGACCGTTG-3'	Real-Time PCR
VSP2-RA	5'-CACGAGACTCTTCCTCACCTTTGA-3'	
JR1-RS	5'-AGTTGCTTGGGATGATGGTGTTC-3'	Real-Time PCR
JR1-RA	5'-CCCATGATCACTTCCAAGGACAG-3'	
JR2-RS	5'-CGTACCAGTGGTTACACTCGGATC-3'	Real-Time PCR
JR2-RA	5'-GAATAGCCGCCTGGATAACTGTC-3'	
LOX2-RS	5'-GAAGAACTGATCACATCGGACGA-3'	Real-Time PCR
LOX2-RA	5'-GAGGTGACCCATGCAATCGTAG-3'	
PR1-RS	5’-AACTACAACTACGCTGCGAACA-3’	Real-Time PCR
PR1-RA	5’-TACACCTCACTTTGGCACATCC-3’	
PR2-RS	5'-CTATTCGACGCAAATCTCGACTCG-3'	Real-Time PCR
PR2-RA	5' -GTCTTTGCGTTTTCCACACTCGTC-3'	
PR3-RS	5'-GTTCTGGATGACTGCTCAGCCTC-3'	Real-Time PCR
PR3-RA	5'-CACTCCAATCCACCGTTAATGATG-3'	
PR5-RS	5’-GGATCGGGAGATTGCAAATAC-3’	Real-Time PCR
PR5-RA	5’-ACGGCAGCAATATTGATCC-3’	
UBQ10-RS	5'-TGACAACGTGAAGGCCAAGATCC-3'	Real-Time PCR
UBQ10-RA	5'-ATACCTCCACGCAGACGCAACAC-3'	
